# Dietary Behaviours, Impulsivity and Food Involvement: Identification of Three Consumer Segments

**DOI:** 10.3390/nu7095379

**Published:** 2015-09-18

**Authors:** Rani Sarmugam, Anthony Worsley

**Affiliations:** 1 Health Promotion Board, 3 Second Hospital Avenue, 168937 Singapore; 2 School of Exercise and Nutrition Sciences, Deakin University, Burwood VIC 3125, Australia; E-Mail: anthony.worsley@deakin.edu.au

**Keywords:** impulsive behaviour, food involvement, fast foods, ready meals, eating behaviour, segmentation

## Abstract

This study aims to (1) identify consumer segments based on consumers’ impulsivity and level of food involvement, and (2) examine the dietary behaviours of each consumer segment. An Internet-based cross-sectional survey was conducted among 530 respondents. The mean age of the participants was 49.2 ± 16.6 years, and 27% were tertiary educated. Two-stage cluster analysis revealed three distinct segments; “impulsive, involved” (33.4%), “rational, health conscious” (39.2%), and “uninvolved” (27.4%). The “impulsive, involved” segment was characterised by higher levels of impulsivity and food involvement (importance of food) compared to the other two segments. This segment also reported significantly more frequent consumption of fast foods, takeaways, convenience meals, salted snacks and use of ready-made sauces and mixes in cooking compared to the “rational, health conscious” consumers. They also reported higher frequency of preparing meals at home, cooking from scratch, using ready-made sauces and mixes in cooking and higher vegetable consumption compared to the “uninvolved” consumers. The findings show the need for customised approaches to the communication and promotion of healthy eating habits.

## 1. Introduction

In Australia, consumers spend about a third (27%) of their average weekly household expenditure on food and non-alcoholic beverages on out-of-home meals (including fast foods). The amount spent on out-of-home meals and fast foods increased by 50% between 2003/2004 and 2009/2010 [1]. In the U.S., the contribution of calories from fast foods increased from 3.1% to 13.2% between 1977–1978 and 2005–2008 [2]. Higher consumption of fast foods is associated with poor dietary quality, higher energy intakes [3,4,5] and higher body weight [3,5,6,7].

In the home setting, the amount of time allocated to meal preparation has decreased in recent years [8,9,10]; suggesting consumers are increasingly relying on convenience or ready meals which require less preparation time. This is consistent with studies which show increasing trends in the energy contributed from ready meals (such as frozen pizza, instant pasta/rice) towards total energy from household food purchases in US and Canada [11,12]. The increased consumption of ready meals is of concern because ready meals tend to have high energy density and poor nutrition quality [13,14] and frequent consumption of these foods are associated with obesity [15].

Changing socio-demographic trends, such as longer working hours [16,17], are among the reasons cited for changing meal preparation behaviours [18]. Long working hours increase the level of stress [19] and fatigue [20] experienced by employees. People who report higher levels of perceived stress and time pressure tend to be more convenience-oriented [21,22,23]; and more likely to consume fast foods on a regular basis [24]. It has also been suggested that under conditions when cognitive processing resources are depleted (*i.e*., low cognitive capacity), behaviour is more likely to be driven by impulsive processes [25].

Impulse buying is a set of automatic behaviours driven by heuristic processes, which represent the interplay between intra-individual psychological factors and environmental cues [26,27]. Experimental studies have shown impulsivity may be associated with higher food intake in normal weight participants compared to those with low levels of impulsivity [28,29]. Impulsiveness also appears to be associated with unhealthy food choices. For example, in a virtual supermarket study, more impulsive, hungry shoppers purchased higher calorie food products [30]. Impulsive consumers tend to make food choices based on taste preferences rather than long term health considerations [31]. Heightened impulsivity is also associated with stronger tendency to overeat in response to external food cues [31].

Although the consumption of meals away from home has increased in the past decade, energy from meals eaten at home accounts for a large part of dietary energy consumption [9,32] confirming the importance of meal preparation at home. Studies of meal preparation, and use of convenience meals (or ready-meals), have consistently shown that in addition to increased stress levels and perceived time pressure, the importance attached to healthy eating and levels of food involvement are often associated with the selection of meal preparation methods [21,33,34].

Food involvement is related to the amount of effort invested in meal preparation. For example, the use of convenience foods or purchase of fast foods requires little effort compared to cooking from scratch. Higher levels of food involvement appear to be associated with healthier dietary behaviour [35,36,37,38], while lower food involvement is associated with higher convenience orientation [33].

Although many studies have linked impulsivity to unhealthy dietary behaviours and food involvement to healthier dietary behaviours; to the authors’ knowledge, no study has attempted to segment consumers using these two predictors and examine their dietary behaviours. Given the importance of automaticity in driving dietary behaviours, such understanding is important to ensure the development of effective health promotion programmes and communication messages.

## 2. Methods

### 2.1. Sample and Procedure

The study population consisted of Australian adults aged 18 years and above. Data were collected through a web-based survey. Survey invitations were sent by email to a market research company’s panel members. The panel members were individuals who had registered and agreed to participate in surveys in return for reward points. The participants were invited to answer a self-administered online questionnaire, which could be completed at their convenience within 20 to 25 min. The survey procedure has been described elsewhere [39]. The study was approved by the University of Wollongong Research Ethics Committee (Ethics reference no: HE11/351).

### 2.2. Questionnaire and Measurement Scales

#### 2.2.1. Psychosocial Measures

Most of the items used in this study were adapted from previous studies. Impulse buying tendency was measured using a scale developed by Verplaken and Herabadi [40]. The original scale consisted of 20 items, which represented two subscales: (1) cognitive processes, which were associated with factors such as lack of planning and deliberation; and (2) affective factors, which were associated with feelings of excitement; lack of control and acting without reflection. The authors have previously demonstrated that the impulse buying measures were related to the Big Five dimensions of personality. The cognitive subscale of the impulse buying tendency scale (e.g., “*most of my purchases are planned in advance*”) was shown to be positively associated with extraversion, and inversely associated with personal need for structure, need to evaluate, and, conscientiousness. The affective subscale was significantly associated with lack of preoccupation, extraversion and negatively associated with autonomy. Ten items with the highest factor loadings or deemed relevant to food shopping were chosen from the original scale; five each from the two subscales: cognitive impulsive factors, and affective impulsive factors.

Nine items from the “preparation and eating” subscale of the food involvement scale developed by Bell and Marshall [6] were used in this survey. Two items were rephrased for this study; “I do most or all of my own food shopping” was changed to “I do most or all of the cooking for my household” according to the objectives of this study, and the item “I do not like to mix or chop food” was changed to “I don’t like preparing food” based on feedback received during pre-testing. Both impulse buying tendency and food involvement were measured using five-point Likert response scales ranging from 1 (strongly disagree) to 5 (strongly disagree).

The importance of eating healthy food was assessed by asking the respondents “How important is it for you to eat healthy food?” using five-point Likert scales ranging from “not important” (1) to “very important” (5). The question structure and response formats were adapted from Watters and Satia [41].

The situational purchasing cues scale was adapted from situational cues items developed by Mihić and Kursan [42]. Only the items related to collateral situational factors (e.g., point of sale advertisements) were used in this study. Based on feedback received from participants during pre-testing of the questionnaire, two additional items, “A new item on the shelf”, and “Saw an item which was advertised on TV/radio/newspaper/website” were considered as situational cues, and therefore were included as part of the measurement of situational purchasing cues. Participants were asked to indicate to what extent each of the situational cues affected their impulse purchases during their food shopping using five-point Likert scales with responses ranging from “not at all” (1) to “a lot” (5).

#### 2.2.2. Dietary Behaviours

Fast food consumption was assessed by two questions: “During the past month, how often did you: (1) “Eat out at fast food restaurants (e.g., McDonalds, KFC, and Pizza Hut)” and, (2) “Have western takeaway meals (e.g., pizza, burgers, fried or roast chicken, McDonalds, KFC)”. In addition, due to the popularity of Asian takeaway meals, participants were asked how often they had consumed Asian takeaway meals (e.g., Chinese, Indian, and Thai) in the past month. A similar question was asked regarding the frequency of preparation or cooking of dinner at home. The items were adapted from those developed by Thornton *et al*. [43].

Three items assessed meal preparation at home and the use of pre-processed products or convenience meals: (1) “Cooked meals from scratch or fresh ingredients”; (2) “Used ready-made sauces, marinades or mixes (e.g., pasta sauce) for cooking” and; (3) “Had ready meals or convenience meals (only requires adding water or heating up)”. These items were based on questions used in previous studies [44,45]. In addition, participants were also asked how frequently they consumed salted snacks.

All the dietary behaviour questions were presented with seven-point response scale options: not applicable or never (1), once a month (2), two to three times per month (3), one to two times per week (4), two to four times per week (5) five or six times per week (6), or daily (7).

Fruit and vegetables consumption was assessed using two items: (1) How many serves of fruit (fresh, frozen or tinned) do you usually eat each day and, (2) How many servings of vegetables (fresh, frozen or tinned) do you usually eat each day? Respondents were asked to indicate the frequency of consumption of fruit and vegetables per day using eight response options: “I don’t eat fruit/vegetables” (1) “less than one serve/day” (2) “1 serve/day” (3) “2 serves/day” (4) “3 serves/day” (5) “4 serves/day” (6) “5 serves/day” (7) and “6 or more serves/day” (8) [46].

### 2.3. Data Analysis

The data were analysed using SPSS Statistics version 20.0 (IBM Corp: Armonk, New York, NY, USA) [47]. Descriptive statistics were used to report frequencies, percentages, means and standard deviations.

Exploratory factor analysis (principal axis factoring with varimax rotation) was used to establish the unidimensionality of the constructs for the impulse buying tendency scale, food involvement scale and situational cues. The number of factors retained was determined by the criterion of eigenvalue greater than 1 and examination of the scree plots. Items with loadings above 0.30 and minimum cross-loadings were retained (see Table 2). Internal reliability values were calculated using Cronbach’s alpha.

Cluster analysis was used to segment the respondents based on their impulse buying and food involvement. Ward’s hierarchical clustering method was employed to determine the number of segments and cluster centroids. This was followed by K-means analysis using cluster centroids from the hierarchical clustering method. The use of the two-stage clustering procedure has been suggested as an option to overcome limitations associated with hierarchical and partitioning clustering methods [48,49,50].

External validation and further profiling were conducted by examining the segments for differences in dietary behaviours and socio-demographic characteristics using analysis of variance (ANOVA) F-tests with Bonferonni and Dunnett’s T3 *post hoc* comparison of mean scores and chi-square tests. This provided information about the characteristics associated with each consumer segment and subsequently the assignment of provisional names to reflect the characteristics of the segment.

## 3. Results

### 3.1. Characteristics of the Study Population

Sample characteristics are shown in Table 1. Slightly more than half of the respondents (58.3%) were female. Similar to the 2006 Australian census data [51], about a third of the respondents (27.0%) had tertiary education. Almost half of the respondents had an annual household income above $60,000.

**Table 1:** Socio-demographic characteristics of the study sample.

		Sample		Census *
		*N*	%	%
**Gender**	Male	221	41.7	48.6 ^†^
	Female	309	58.3	51.4
**Age (years)**	18–20	15	2.8	3.6 ^†^
	21–30	82	15.5	17.6
	31–40	84	15.8	19.5
	41–50	96	18.1	19.6
	51–60	85	16.0	16.8
	61–70	123	23.2	11.1
	>70	45	8.5	11.8
**Highest level of education**	Left school at 16 or 18 years	217	40.9	
	Technical and Further Education (TAFE) or college diploma, certificate or formal trade qualification	170	32.1	45.4 ^‡,^^§,^^**║**^
	Bachelor degree/Graduate Diploma/Graduate Certificate	104	19.6	24.5
	Postgraduate degree	39	7.4	4.9
**Employment**	Employed full-time	175	33.0	36.6 ^‡,^^§,^^¶^
	Employed part-time/casual	93	17.5	16.9
	Home duties/retired/student	212	40.0	33.1
	Unemployed/looking for work	50	9.4	3.2
**Household income (AUSD)**	10,000 or less	26	4.9	
	10,001 to 20,000	63	11.9	
	20,001 to 40,000	96	18.1	
	40,001 to 60,000	94	17.7	
	60,001 to 80,000	81	15.3	
	80,000 to or 100,000	71	13.4	
	Over 100,001	99	18.7	

* Based on 2006 Census data [51]; ^†^ Based on census data for population aged 18 and above; ^‡^ Based on individuals aged 15 years and over who stated completed qualification; ^§^ Denotes slight variation of categories between survey and census; **^║^** Total percentages do not add up to 100% due to individuals who did not state or inadequately their level of education; ^¶^ Total percentages do not add up to 100% due to individuals who did not state their employment status.

### 3.2. Exploratory Factor Analysis

Exploratory factor analyses of the impulse buying tendency and food involvement items revealed two-factor solutions, which explained 45.32% and 39.92% of the total variance in the data, respectively. Similar to the scale developed by Verplanken and Herabadi [40], the two impulse buying tendency constructs derived here are referred to as “Impulse buying (affective)” and “Impulse buying (cognitive)”.

The first food involvement construct represents items that are directly related to cooking and food preparation. Therefore, this construct was labelled as “Food involvement (meal preparation)”. The second food involvement construct consists of items that are related to the importance of food in different scenarios (e.g., daily food decision, eating out, travel). This construct was labelled as “Food involvement (importance of food)”.

Factor analysis of the sensitivity to situational cue items derived one factor, which explained over 41.83% of the total variance. The Cronbach’s alpha values for four out of the five factors were above 0.7, while one (food involvement (hedonism)) was slightly above 0.6 (Table 2), which indicates that all the factors had an adequate level of reliability or internal consistency [52]. Factor loadings and reliability coefficients for all the measurement items are presented in Table 2. Scores for the items in each construct were summed and used to represent the construct in subsequent analyses.

**Table 2:** Factor loadings and reliability estimates for measurement items.

Construct/Item	Factor Loadings
**Impulse buying (affective)** *Cronbach’s alpha:* 0.78	
It is a struggle to leave nice things I see in a shop	0.81
I sometimes cannot suppress the feeling of wanting to buy something	0.68
If I see something new, I want to buy it	0.55
I sometimes feel guilty after having bought something	0.55
I find it difficult to pass up a bargain	0.53
**Impulse buying (cognitive)** *Cronbach’s alpha:* 0.71	
I usually only buy things that I intended to buy *	0.85
I only buy thing I really need *	0.69
Most of my purchases are planned in advanced *	0.46
**Food involvement (meal preparation)** *Cronbach’s alpha:* 0.83	
I don’t like preparing food *	0.84
I enjoy cooking for others and myself	0.79
Cooking or barbequing is not much fun *	0.63
**Food involvement (importance of food)** *Cronbach’s alpha:* 0.61	
I don’t think much about food each day *	0.63
When I eat out, I don’t think or talk much about how the food tastes *	0.48
Talking about what I ate or am going to eat is something I like to do	0.47
Compared with other daily decisions, my food choices are not very important	0.45
When I travel, one of the things I anticipate most is eating the food at the place I visit	0.31
**Sensitivity to situational cues** *Cronbach’s alpha:* 0.83	
Special product arrangement or display (e.g., chocolate at end of aisle bin)	0.72
Saw an item which I had seen advertised on TV/Radio/Newspaper/Website advertisement	0.70
Point-of-sale advertisements (*i.e*., at the store), fliers or notices	0.68
New item on the shelf	0.67
Point-of-sale events (e.g., cooking demonstration or free tasting)	0.59
Shelf arrangement (e.g., products within hand reach)	0.58
Promotional activities (e.g., buy 1 and get 2, 50% off)	0.58

* items were reverse coded.

### 3.3. Segmentation Analysis

Cluster analysis based on impulsive buying tendency and food involvement resulted in a three-segment solution. Descriptive analysis of the mean scores of the segments and ANOVA analysis shows the consumers in first segment were characterised by high scores for both constructs of impulsivity, and food involvement (importance of food). Therefore, this segment was named as the “impulsive, involved” segment. The consumers in the second segment had lower scores for both impulsivity constructs and higher levels of food involvement compared to the consumers’ in the third segment. Examination of the dietary behaviours of the consumers in this segment shows their dietary behaviours were healthier compared to others (Table 3). Therefore, this segment was labelled as the “rational, health conscious” segment. The consumers in the third segment reported the lowest levels of food involvement compared to the other segments and intermediate levels of impulsive buying tendency scores. This segment was named as the “uninvolved” segment (Table 4).

**Table 3:** Dietary behaviours and meal preparation practices of the segments.

	The Impulsive, Involved (*n* = 177)	The Rational, Health Conscious (*n* = 208)	The Uninvolved (*n* = 145)	
	**(%)**	**(%)**	**(%)**	**F**
**Eating out and takeaways**				
Fast food restaurants (mean ± SD)	2.26 (1.20) ^b^	1.68 (1.96) ^a,c^	1.96 (1.10) ^a^	13.57 ***
*Never/NA*	35.6	55.8	49.0	
*1–3 times/month*	43.5	39.4	38.6	
*Once a week or more*	20.9	4.8	12.4	
Western takeaway meals (mean ± SD)	2.33 (1.21) ^b^	1.84 (1.00) ^a,c^	2.14 (1.15) ^b^	9.56 ***
*Never/NA*	29.4	48.1	39.3	
*1–3 times/month*	52.5	45.7	44.8	
*Once a week or more*	18.1	6.3	15.9	
Asian takeaway meals (mean ± SD)	1.93 (1.01) ^b^	1.46 (0.70) ^a^	1.67 (0.93) ^a^	13.71 ***
*Never/NA*	40.1	63.9	56.6	
*1–3 times/month*	52.5	33.7	37.9	
*Once a week or more*	7.3	2.4	5.5	
**Use/Consumption of processed foods**				
Used ready-made sauces, marinades or mixes (mean ± SD)	3.21 (1.32) ^b,c^	2.88 (1.33) ^a^	2.81(1.32) ^a^	4.52 *
*Never/NA*	10.7	22.1	23.4	
*1–3 times/month*	45.8	43.3	45.5	
*Once a week or more*	43.5	34.6	31.0	
Had ready meals or convenience meals (mean ± SD)	2.25 (1.37) ^b^	1.74 (1.15) ^a,c^	2.08 (1.23) ^b^	8.25 ***
*Never/NA*	43.5	63.0	45.5	
*1–3 times/month*	36.7	26.9	37.9	
*Once a week or more*	19.8	10.1	16.6	
Salted snacks (e.g., potato/corn chips) (mean ± SD)	3.27 (1.46) ^b^	2.52 (1.39) ^a,c^	3.08 (1.67) ^b^	13.06 ***
*Never/NA*	15.3	32.7	24.8	
*1–3 times/month*	40.7	39.9	33.1	
*Once a week or more*	44.1	27.4	42.1	
**Meal preparation behaviours**				
Prepared meals at home (mean ± SD)	5.67 (1.26) ^c^	5.81 (1.32) ^c^	5.14 (1.70) ^a,b^	10.04 ***
*Never/NA*	1.7	3.8	6.9	
*1–3 times/month*	5.6	1.9	9.0	
*1–4 times/week*	23.2	19.2	28.3	
*5 times/week or more*	69.5	75.0	55.9	
Cooked meals from scratch or fresh ingredients (mean ± SD)	5.28 (1.48) ^b,c^	5.66 (1.39) ^c^	4.59 (1.90) ^a,b^	19.96 ***
*Never/NA*	4.0	3.8	13.8	
*1–3 times/month*	6.8	3.8	9.7	
*1–4 times/week*	37.3	23.1	36.6	
*5 times/week or more*	52.0	69.2	40.0	
Used herbs and spices as flavouring (mean ± SD)	5.02 (1.46) ^c^	4.88 (1.78) ^c^	3.99 (1.82) ^a,b^	16.86 ***
*Never/NA*	2.3	8.7	15.9	
*1–3 times/month*	12.4	12.0	16.6	
*1–4 times/week*	46.9	34.1	46.2	
*Once a week or more*	38.4	45.2	21.4	
**Consumption of fruit and vegetables**				
Fruit (mean ± SD)	3.38 (1.18)	3.48 (1.32)	3.19 (1.27)	2.27
*Don’t eat fruit/1 servings or less*	52.0	53.4	62.8	
*2–3 servings*	45.2	39.4	33.1	
*4 servings and more*	2.8	6.3	4.1	
Vegetables (mean ± SD)	4.28 (1.46) ^c^	4.39 (1.48) ^c^	3.74 (1.50) ^a,b^	8.99 ***
*Don’t eat vegetables/1 servings or less*	32.8	30.3	50.3	
*2–3 servings*	47.5	51.4	38.6	
*4 servings and more*	19.8	18.3	11.0	

*** *p* < 0.001; * *p* < 0.05; ^a^ Significant difference between the “rational, health conscious” and “impulsive, involved”; ^b^ Significant difference between the impulsive, involved” and “rational, health conscious”; ^c^ Significant difference between “impulsive, involved” and “uninvolved”.

**Table 4:** Segment and profiling variables.

	The Impulsive, Involved (*n* = 177)		The Rational, Health Conscious (*n* = 208)		The Uninvolved (*n* = 145)		
**Size of segment (%)**	33.4		39.2		27.4		
**Segmentation variables**	**Mean**	**SD**	**Mean**	**SD**	**Mean**	**SD**	**F**
Impulsive buying tendency (affective)	16.74 ^b,c^	2.36	9.90 ^a,c^	2.29	13.64 ^a,b^	2.88	364.74 ***
Impulsive buying tendency (cognitive)	8.44 ^b,c^	2.24	6.85 ^a,c^	2.08	7.86 ^a,b^	2.02	27.71 ***
Food involvement (importance of foods)	18.60 ^b,c^	2.30	16.91 ^a,c^	2.56	13.91 ^a,b^	2.25	155.05 ***
Food involvement (meal preparation)	11.40 ^c^	2.05	11.36 ^c^	2.05	7.32 ^a,b^	2.34	189.71 ***
**Descriptor variables**							
Importance of eating healthy foods	4.15 ^c^	0.79	4.17 ^c^	0.68	3.86 ^a,b^	0.93	8.07 ***
Situational cues	17.10 ^b,c^	5.29	12.16 ^a,c^	4.05	13.70 ^a,b^	4.51	55.78 ***

*** *p* < 0.00; ^a^ Significant difference between the “rational, health conscious” and “impulsive, involved”; ^b^ Significant difference between the impulsive, involved” and “rational, health conscious”; ^c^ Significant difference between “impulsive, involved” and “uninvolved”.

The following sections describe the psychosocial, dietary behaviours and socio-demographic characteristics of each consumer segment in detail.

**Segment 1: The impulsive, involved consumers**

The “impulsive, involved” segment was the largest group of consumers (41.5%). They were characterised by their high level of involvement with all food-related experiences. This segment reported the highest levels of impulsive buying tendency (both affective and cognitive), and higher importance attached to food measures of food involvement compared to the other two consumer segments. In addition, they were more likely to report that their impulsive purchases during food shopping were strongly influenced by situational cues compared to those of other segments (Table 4).

The segment’s greater impulsivity and food involvement are reflected in their reported dietary practices. For example, they reported significantly more frequent consumption of fast foods, take-aways, convenience meals and salted snacks compared to the “rational, health conscious” segment. About one-fifth of the segment members consumed fast foods or used convenience meals or ready meals at least once a week. These “impulsive, involved” consumers also reported the most frequent use of ready-made sauces and mixes in their cooking compared to the other two segments.

Consistent with their higher levels of involvement in meal preparation, this group of consumers reported greater frequencies of preparing meals at home, cooking from scratch and using herbs and spices in cooking compared to the “uninvolved”. They also attached higher levels of importance to healthy eating compared to the “uninvolved” segment. Accordingly, their intakes of fruit and vegetables were higher than those of the “uninvolved” (Table 3).

Compared to the total sample, the segment included relatively higher proportions of younger adults, females and those who were either employed full time or part-time. More consumers in this segment also reported household incomes above $80,000 compared to the other two segments. More of the “impulsive, involved” consumers also reported living in larger households and more of them lived with children below 18 years (Table 5).

**Table 5:** Socio-demographic characteristics of the segments.

	The Impulsive, Involved (*n* = 177)	The Rational, Health Conscious (*n* = 208)	The Uninvolved (*n* = 145)	χ^2^ (d*f*)
	**(%)**	**(%)**	**(%)**	
**Gender**				11.07(2) **
*Female*	68.4	53.4	53.1	
**Age** (mean ± SD)	43.5 ± 16.8	54.0 ± 15.8	49.2 ± 15.3	
*≤30*	29.4	11.5	14.5	4.29(4) ***
*31-60*	50.3	46.2	55.2	
*≥61*	20.3	42.3	30.3	
**Education**				
*Left school at 16/18 years*	43.5	37.0	43.4	2.96(4)
*Technical and Further Education (TAFE) or college diploma, certificate or formal trade qualification*	29.9	33.2	33.1	
*Bachelor degree/Graduate Diploma/Graduate Certificate/Postgraduate*	26.6	29.8	23.4	
**Employment**				14.04(6)*
*Employed full-time*	37.3	30.8	31.0	
*Employed part-time/casual*	23.7	13.5	15.9	
*Home duties/retired/student*	30.5	45.2	44.1	
*Unemployed/looking for work*	8.5	10.6	9.0	
**Household income**				14.92(4) **
*$40,000 and below*	26.0	40.4	37.9	
*$40,001 to $80,000*	33.9	28.8	37.9	
*Over $80,000*	40.1	30.8	24.1	
**No of people in household**				18.58 **
*Single/2 people*	44.1	62.5	55.9	
*3-4 people*	41.2	32.2	37.2	
*>4 people*	14.7	5.3	6.9	
**No of children below 18 years**				10.55
*None*	66.9	77.7	75.9	
*One*	14.5	13.1	12.8	
*Two*	11.6	7.3	8.5	
*Three and more*	7.0	1.9	2.8	
**Presence hypertension, heart disease or stroke**				
*Yes*	20.3	31.3	20.0	8.36 *
**Cooking for the household**				9.81(4) *
*Main role*	67.8	63.0	57.9	
*Share responsibility*	24.3	28.4	24.8	
*Do not cook*	7.9	8.7	17.2	

***: *p* < 0.001; **: *p* < 0.01; *: *p* < 0.05.

**Segment 2: The rational, health conscious consumers**

The “rational, health conscious” segment included 39.2% of the sample. This segment reported the lowest levels of impulse buying tendency and sensitivity to situational cues during their impulse purchases compared to the other segments. They also reported significantly higher levels of food involvement in meal preparation and placed higher levels of importance on eating healthy foods than the “uninvolved” (Table 4).

The segment consisted of consumers who were the least likely to engage in unhealthy dietary practices and most likely to engage in healthier dietary practices. For example, they reported significantly lower frequencies of eating takeaways from fast-foods restaurants and using convenience or ready-meals compared to other two consumer segments.

The “rational, health conscious” consumers were most likely to prepare meals at home and from scratch. Three quarters (75%) of them prepared their own meals at least five times or more a week. Although a high proportion (70%) of the “impulsive, involved” segment also reported preparing meals at home at least five times or more a week, almost 70% of the “rational, health conscious” reported preparing meals from scratch (an indication of healthier meal preparation) compared to the 52% of the “impulsive, involved”. Similarly, their use of ready-made sauces, marinades or mixes was significantly lower than the “impulsive, involved” segment. They also reported greater consumption of vegetables compared to the “uninvolved” and the lowest consumption of salted snacks compared to other two segments (Table 3).

The segment included older consumers than the other segments and almost one third had completed tertiary education. Consistent with their greater age, fewer of them were in full or part-time employment and more of them lived in smaller households (alone or with another person), had no children below 18 years living them or had been diagnosed with hypertension, heart disease or stroke (Table 5).

**Segment 3: The Uninvolved Consumers**

The “uninvolved” consumers were the smallest segment (22.6%). The members of this segment were characterised by their indifference to meal preparation and healthy eating (Table 4). Consistent with their low levels of food involvement and importance attached to healthy eating, this segment reported the lowest frequencies of behaviours associated with meal preparation at home including cooking meals from scratch, and using herbs and spices in cooking. In comparison to other segments, a sizeable proportion (15.9%) of the “uninvolved” did not cook or cooked less than three times a month. They also reported the lowest consumption of vegetables (Table 3).

The levels of impulsivity and hedonism of the “uninvolved” were lower than the “impulsive, involved” consumers but higher than the “rational, health conscious” consumers. This reflects the differences observed in their reported intakes of “unhealthy foods”. For example, their consumption of fast foods, convenience meals and salted snacks were lower than the “impulsive, involved” but higher than the “rational, health conscious”.

In comparison to the other segments, the “uninvolved” segment included fewer individuals with incomes in the higher income category and more individuals without a tertiary degree. While the gender, and employment distribution of this segment was similar to the “rational, health conscious” segment, only 20% of the “uninvolved” consumers been diagnosed with hypertension, heart disease or stroke as compared with a third of the rational, health conscious” segment.

## 4. Discussion

This study demonstrates the utility of psychographic variables, specifically impulsivity and food involvement, in consumer segmentation analysis. Importantly, the study showed that consumers who were more impulsive (the “impulsive, involved”) and the moderately impulsive (“uninvolved”) tended to consume fast foods, ready meals or convenience meals and salted snacks more frequently than the “rational, health conscious” segment.

The unhealthy dietary practices of the more impulsive consumers are supported by previous studies which have shown associations between higher levels of impulsivity with higher calorie intake [53,54], and higher snack consumption [30]. Consumers in these two segments (the “impulsive, involved” and the “uninvolved”) also reported being more influenced by situational cues in their impulse purchases than those in the “rational, health conscious” segment. Indeed, situational cues can serve as external triggers to impulsive behaviours or impulses [55,56] and consumers with higher impulsivity appear more likely to be sensitive to external situational cues such as advertisements and promotional gifts [56].

Although higher levels of food involvement tend to be associated with healthier dietary behaviour including higher consumption of fruit and vegetables [35,36,37,38], in this study, consumers with higher levels of involvement may not have practiced healthier dietary habits exclusively. For example, the “impulsive, involved” segment which reported higher levels of food involvement as well as impulsivity were characterised by involvement in all food-related experiences which includes both healthier and less healthy dietary practices.

This may reflect the inter-play between the rational and automatic processes which underpin dietary behaviours which may change according to usage situations [57]. For example, after a long day at work, given their higher levels of impulsiveness, consumers in this segment may be more inclined to rely on quick-solutions and find it easier to pick-up take-ways or fast foods on the way home, or heat-up a meal instead of cooking from scratch. It is also possible that the different aspects of involvement, for example, the task-oriented aspects of food involvement (e.g., cooking) *vs.* consumers’ affective relationships with food (e.g., the perceived importance of food) exert different influences on eating behaviour.

Although there was no significant difference between the levels of importance attached to healthy eating between the two segments, the “impulsive, involved” segment consumed higher amounts of fast foods, convenience meals and snacks than the “rational, health conscious” segment. Such inconsistencies observed in consumer behaviours are not uncommon [58]. This may be because food choices are influenced by a variety of factors, such as taste, nutritional value, cost and convenience [59,60], and the importance of these factors vary for each individual. For most people, taste and cost are more important than nutrition [59], and consumers who might be aware of the long term consequences of eating fast foods may still consume them because of the influence of factors such as taste [61], or the need for immediate gratification [62].

Given the profile of the “impulsive, involved” consumers, it is possible their behaviour is driven more by impulsivity, hedonism and immediate gratification while, the “rational, health conscious” who were older and more likely to be tertiary educated and affected by chronic disease, compared to the “impulsive, involved” may be driven by the health and nutrition value of food.

Several demographic characteristics associated with the segment profiles are also consistent with other studies. For example, higher proportions of younger adults appear in unhealthy lifestyles consumer segments [58,63]. As in this study, past studies have shown that highly educated consumers have greater involvement with food [35,37] possibly because they enjoy eating, and place higher priority on cooking in their lives [35,37].

Although higher levels of education and income are often associated with healthier dietary behaviours [64], in this study, the two consumer segments which reported higher consumption of convenience meals included consumers with higher levels of income (“impulsive, involved”) as well as those with low incomes (“uninvolved”). This apparent contradiction is similar to that found in an earlier segmentation study which also showed that consumers from both low and high income backgrounds were frequent users of convenience foods [65]. It is plausible that this is because time pressure or time-scarcity affects both, high-income groups, as well as low-income groups [66,67]. Consumers from low income groups may opt for convenience meals as a strategy to cope with role overload [68] while consumers with higher incomes may rely on convenience meals because they place greater value on leisure time [65,69].

### 4.1. Recommendations for Health Promotion Practice

Since the “impulsive, involved” and the “uninvolved” exhibited greater impulsivity and poorer dietary practices, they might be targeted in future health promotion programmes. Impulsive consumers tend to be more sensitive to situational cues that they commonly encounter in the food shopping environment [56]. Marketers have generally taken advantage of this relationship to increase product sales. Similarly, health promoters might work towards identifying the triggers and types of purchases made by consumers who are prone to impulse buying to plan more effective health promotion campaigns.

Differences in the levels of food involvement between the segments indicate a need for targeted communication strategies because individuals with higher levels of involvement may process information differently compared to those with low levels of involvement [70]. For example, people with low levels of interest in food (such as the “uninvolved” segment) are unlikely to pay attention to any information about food and health. Therefore, instead of traditional communication strategies which provide strong rational arguments, health promoters should consider reaching out to this segment using communication strategies which can be processed via peripheral routes of persuasion, for example via manipulation of environmental cues [71]. Conversely, given their higher levels of food involvement and importance attached to healthy eating, the “impulsive, involved” segment is more likely to be receptive to health and food related messages. Therefore, health promoters might consider more complex, persuasive rational arguments in formulating communication strategies to appeal to this segment of consumers.

The present findings differentiate between meal preparation practices among different segments of consumers. Although both, the “impulsive, involved” and the “rational, health conscious” segments reported higher frequencies of meal preparation at home, the “rational, health conscious” segment appears to have practiced healthier home meal preparation, cooking meals from scratch while the “impulsive, involved” segment opted for “quick solutions” and relied on ready-made sauces and mixes when cooking at home. The differences in meal preparation may have significant impacts on consumers’ health. Processed foods such as convenience meals and ready-made sauces tend to be higher in energy [72] and sodium [73].

The differences observed in meal preparation behaviours show the need for more targeted and realistic communication messages. For example, it may be more practical to teach those consumers who are more impulsive and involved how to incorporate healthier processed foods, such as canned and frozen vegetables and beans, in cooking instead of asking them to cook from scratch. In addition, health promoters might also consider equipping the consumers in this segment with meal planning skills which may help them to avoid or reduce their reliance on convenience and pre-cooked meals [74].

The population segments identified through the cluster analysis could potentially be reached through collaborative partnerships in order to promote healthier dietary behaviours. For example, nutrition promoters could explore collaborations with local supermarkets and use choice architecture or in-store marketing cues to influence the purchasing behaviours of consumers [75]. More specifically, this could be used to influence the “impulsive, involved” and the “uninvolved” consumers who are more attentive and susceptible to environmental cues such as product sampling and store displays (e.g., end-of-aisle displays, store windows). In addition, health promoters may also consider “budget-friendly” initiatives to appeal to the “uninvolved”, which consists of a higher proportion of those with lower incomes and who may therefore be more cost-conscious. Some activities that can be considered include working with retailers to highlight cheaper and healthier food products in the supermarkets.

The “impulsive, involved” segment relied on convenience meals and ready-made sauces and mixes when cooking at home. This could be because of a lack of cooking skills [76]. Given the high levels of food involvement, and importance placed on health, the “impulsive, involved” consumers might be more receptive towards initiatives, which impart cooking skills and make healthy eating more exciting. For example, policy makers might initiate collaboration with popular cooking programmes on television, such as Master Chef [77], to tap the popularity of the programme to disseminate cooking skills and healthy recipes.

Health promoters may also consider promoting healthy eating by reducing the barriers that prevent the adoption of healthier behaviours. For example, the “impulsive, involved” segment contained more consumers with children below 18 years compared to other segments. Assistance from youth based organisations with the completion of children’s homework during after-school care has been shown to be useful in reducing the time constraints often faced by mothers; thus, allowing them to prepare dinner at home [78]. Similar partnerships between community organisations and health promoting agencies should be explored to promote adoption of healthier meal preparation practices.

This study shows the need for customised approaches in health promotion programmes taking into consideration psychosocial factors, such as consumer impulsivity and food involvement. Although health promotion programmes can be delivered without the use of segmentation, such approaches assume that all consumers have similar needs and require similar communication approaches [79,80]. This can be ineffective and costly. Segmentation allows health promoters to identify key groups of consumers and to prioritise resources based on factors such as consumer receptivity, size and risk exposure of segments. The decision to select the segment to be prioritised should be made by health promoters based on their objectives and availability of resources.

### 4.2. Limitations

This study has several limitations. First, the survey used a market research company’s research panel to derive a quota sample, not a random probability sample. Therefore, the generalisability of the findings to the general population is uncertain. However, the sample appears to be more representative of the general population than samples from other surveys in which female respondents or those with tertiary education are often over-represented [81,82]. In the present sample, the proportions of female respondents and those with tertiary education were similar to those in the general population [51].

Second, all the dietary behaviours were measured using frequencies of practice and consumption of these behaviours (for example, frequency of eating fast foods), and no information was gathered on the quantity of food consumed and the quality of individuals’ diets [83]. It is possible that respondents made healthier choices during their purchase or consumption. Similarly, meals prepared at home, or from scratch can also be unhealthy. Thus, the results should be carefully interpreted.

Third, due to the self-administrative mode of the survey, the survey had to be simple and straight forward to complete [84]. Thus, the decision was made to reduce the number of items used to represent several constructs and response scales (*i.e*., impulse buying tendency, food involvement and sensitivity to situational cues) compared to their original form. However, the reliability analyses showed that all these short scales demonstrated satisfactory internal consistency (Cronbach’s alpha more than 0.6).

Fourth, this examined only two segmentation factors; impulsivity and food involvement. It is possible that environmental factors such as time pressure and stress may heighten impulsiveness and so contribute to poor dietary behaviours. Further research is required to examine how the three consumer segments may perceive and respond to different health promotion messages and tools.

Finally, cluster analysis is one of several approaches that can be used to examine this complex data set. One complementary approach is structural equation modelling which is described elsewhere [85].

## 5. Conclusions

This study identified three consumer segments; the “impulsive, involved”, “rational, health conscious” and “uninvolved”. The “impulsive, involved” and the “uninvolved” segments reported more frequent consumption of fast foods, ready meals or convenience meals and salted snacks than the “rational, health conscious” segment, and therefore should be targeted for future health promotion intervention strategies. The segmentation suggests several ways to guide the conceptualisation and implementation of targeted communication strategies according to the likely receptiveness of the consumer segments. Such targeted initiatives are required to promote healthy eating in the current environment where consumers are exposed to extensive food marketing and advertising.
